# Exposure to Neighborhood Walkability and Residential Greenness and Incident Fracture

**DOI:** 10.1001/jamanetworkopen.2023.35154

**Published:** 2023-09-28

**Authors:** Zhanghang Zhu, Zongming Yang, Lisha Xu, Yonghao Wu, Luhua Yu, Peng Shen, Hongbo Lin, Liming Shui, Mengling Tang, Mingjuan Jin, Jianbing Wang, Kun Chen

**Affiliations:** 1 Department of Public Health, Second Affiliated Hospital, Zhejiang University School of Medicine, Hangzhou, China; 2 Department of Public Health, and Department of National Clinical Research Center for Child Health, The Children’s Hospital, Zhejiang University School of Medicine, Hangzhou, China; 3 Department of Chronic Disease and Health Promotion, Yinzhou District Center for Disease Control and Prevention, Ningbo, China; 4 Yinzhou District Health Bureau of Ningbo, Ningbo, China; 5 Department of Public Health, Fourth Affiliated Hospital, Zhejiang University School of Medicine, Hangzhou, China

## Abstract

**Importance:**

Emerging studies have suggested that environmental factors are associated with fracture. However, little is known about the association of neighborhood walkability and residential greenness with fracture.

**Objective:**

To investigate the association of long-term exposure to walkability and greenness with incident fracture and explore the potential interaction effect.

**Design, Setting, and Participants:**

This cohort study recruited participants aged 40 years or older in Ningbo, China from June 2015 to January 2018. Participants were observed for outcomes through February 2023, with data analysis conducted in March 2023.

**Exposures:**

Neighborhood walkability was measured by a modified walkability calculation method according to a walk score tool. Residential greenness was assessed by satellite-derived normalized difference vegetation index (NDVI) within a 1000-m buffer.

**Main Outcomes and Measures:**

Incident fracture was ascertained according to *International Statistical Classification of Diseases and Related Health Problems, Tenth Revision* codes via the Yinzhou Health Information System. Cox proportional hazards models were fit, with age as time scale to estimate the associations of walkability and greenness with fracture. Potential effect modification was explored by covariates, as well as the interactive effect of walkability and greenness.

**Results:**

A total of 23 940 participants were included in this study with 13 735 being female (57.4%). The mean (SD) age at baseline was 63.4 (9.4) years. During a follow-up period of 134 638 person-years, 3322 incident fractures were documented. In the full adjusted model, every IQR increment in neighborhood walkability and residential greenness was associated with a hazard ratio (HR) of 0.88 (95% CI, 0.83-0.92) and 0.84 (95% CI, 0.80-0.89), respectively, for fracture. Furthermore, the association of greenness and fracture was greater with an increase in walkability. The HR (Q4 vs Q1) for greenness was 0.62 (95% CI, 0.46-0.82) in neighborhoods with the highest quartile of walkability.

**Conclusions and Relevance:**

This population cohort study suggested that long-term exposure to neighborhood walkability and residential greenness were both associated with lower risk of incident fracture. The benefits of greenness increased in more walkable areas.

## Key Points

**Question:**

Are neighborhood walkability and residential greenness associated with decreased risk of incident fracture?

**Findings:**

In this cohort study of 23 940 adults aged 40 years or older, higher neighborhood walkability and living in greener areas were associated with decreased risk of fracture.

**Meaning:**

These findings suggest increasing walkability and greenness may be associated with improvement of fracture, emphasizing the importance of creating neighborhoods that offer ample opportunities for exposure to green space, along with a design that supports easy access to key services and destinations to prevent fracture.

## Teaser

This cohort study evaluates the association between incident fracture and exposure to neighborhood walkability and residential greenness.

## Introduction

Fracture has been an important global health issue and has caused a considerable number of disabilities and mortalities worldwide.^1,2^ According to the Global Burden of Disease report of 2019,^3^ approximately 178 (95% CI, 162-196) million new cases of fracture occurred and the years lived with disability were 25.8 million (95% CI, 17.8-35.8). It is projected that the incidence of fracture among people aged 50 years or older in China will increase from 7.15 per 1000 people in 2010 to 9.84 per 1000 people in 2050, with costs projected to increase from $9.45 billion to $25.43 billion.^4^

Over the past decade, several environmental factors have been reported to be associated with the incidence of fracture.^5^ With the progress of urbanization, the share of the global urban population will increase from 56% in 2021 to 68% by 2050.^6^ Therefore, the importance of exploring the role of the built environment on human health has been stressed. One such indicator is neighborhood walkability, reflecting the extent to which the neighborhood area is friendly for pedestrians.^7^ Several studies^8,9,10,11^ have shown that people residing in more walkable neighborhoods may experience more psychological well-being and social connection and engage in more minutes of moderate to vigorous physical activity.

Another environmental factor that may impact fracture is greenness, which is believed to confer benefits such as providing opportunities for physical activity, mitigating hazards of air pollution, increasing social contact and alleviating psychophysiological stress.^12,13,14,15^ Studies have demonstrated that increased physical activity is associated with a decreased risk of fracture.^16,17,18^ Long-term exposure to air pollution is inversely associated with bone health, such as diminished bone density, increased risk of osteoporosis and greater susceptibility to fracture.^19,20,21,22^ In light of these findings, it is crucial to investigate the association between greenness and incident fracture and to prioritize efforts to create healthier environments for residents.

However, to date, limited studies have been available to investigate the association of greenness and walkability with fracture. To our knowledge, only 2 studies have evaluated the relationship between greenness and fracture, and the results remain inconsistent. A cross-sectional study reported that living in greener areas may be associated with higher bone strength and decreased risk of fracture,^23^ whereas a prospective cohort study in China reported higher risk of incident fracture among people living near higher levels of green space.^24^ No prior studies have assessed the association of neighborhood walkability with fracture.

Herein, we aimed to investigate the association of neighborhood walkability and residential greenness with incident fracture using a population-based cohort study. We further evaluated the joint association and interaction effect of walkability and greenness. We hypothesized that greater neighborhood walkability and residential greenness would be associated with lower risk of incident fracture.

## Methods

### Study Population

**Figure 1.**
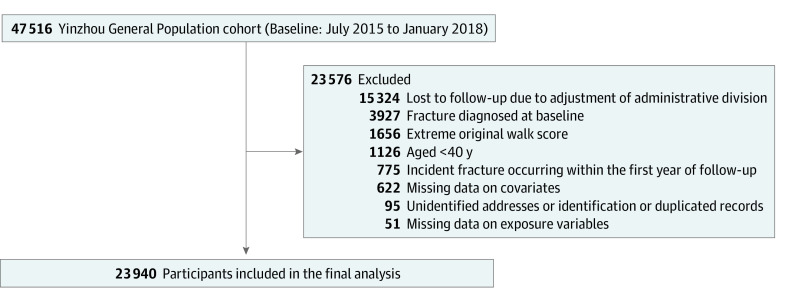
Flow Diagram of the Study Population

This study was conducted based on a prospective cohort in the Yinzhou District of Ningbo, which is located in the eastern coastal area of China. The detailed recruitment process has been described elsewhere.^25^ Briefly, we recruited participants aged older than 18 years from June 2015 to January 2018. In the present study, we excluded participants lost to follow-up due to administrative adjustment, with unidentified addresses, unidentified identification, or duplicated records, and with a fracture diagnosis before baseline. We also excluded participants aged younger than 40 years, participants with a fracture diagnosis within 1 year after baseline, participants with missing data on exposure variables and covariates, and participants with extreme original walk scores (outliers: <Quartile [Q] 1−1.5 × [Q3−Q1] or >Q3 + 1.5 × [Q3−Q1]).^26^ The process of participant selection is shown in Figure 1. All participants provided written informed consent. Ethical approval was provided by the institutional review board of Zhejiang University School of Medicine. This study conformed to the Strengthening the Reporting of Observational Studies in Epidemiology (STROBE) reporting guideline.

### Assessment of Exposures and Covariates

Neighborhood walkability and residential greenness were measured by a modified Walk Score tool and satellite-derived Normalized Difference Vegetation Index (NDVI) (eMethods in Supplement 1). Data on age, sex, body mass index (BMI), education, occupation, household income, history of osteoporosis, history of diabetes, and concentration of particulate matter with a diameter less than 2.5 μm (PM_2.5_) were measured at baseline (eMethods in Supplement 1).

### Follow-Up and Outcome

Yinzhou Health Information System (YHIS) covers all health service institutions (general hospitals, community health service centers, and community health service stations) and an integrated chronic disease surveillance system, a hospital information system, death registry records, and a regional medical insurance system for almost 98 percent of permanent residents in the Yinzhou district.^27,28^ Incident fracture that occurred after the baseline and before the end of follow-up (February 28, 2023) were ascertained via linkage to the YHIS. *International Statistical Classification of Diseases and Related Health Problems, Tenth Revision (ICD-10) *was used to ascertain incident fracture during the follow-up period. Participants who had at least 1 medical record with *ICD-10* codes (S02, S12, S22, S32, S42, S52, S62, S72, S82, and S92) were defined as fracture cases. The number of fractures by different sites and *ICD-10* codes is presented in eTable 1 in Supplement 1. The date of first diagnosis was regarded as incident date.

### Statistical Analysis

Baseline characteristics were examined by calculating the mean (SD) or median (IQR) for continuous variables and the proportions for categorical variables. We also calculated Spearman correlations between walkability and greenness.

Cox proportional hazards models, with age as the underlying time scale, were used to estimate the associations of neighborhood walkability and residential greenness with incident fracture. We modeled associations for walkability and greenness as follows: (1) model 1, adjusted for age (specified as the underlying time scale); (2) model 2, model 1 plus sex, household income, education, occupation, BMI, history of osteoporosis, history of diabetes, and concentration of PM_2.5_; (3) model 3, model 2 plus another exposure variable (greenness or walkability). Results were expressed as hazard ratios (HRs) and 95% CIs per IQR increase in each of the upper 3 Qs relative to the lowest Q. We assessed the proportional hazards assumption for variables included in our models by evaluating the weighted Schoenfeld residuals (all *P* > .05). We used restricted cubic spline (RCS) models with 3 knots to examine exposure–response association.

Assuming additive effects of joint exposures, we used the cumulative risk index (CRI) method to calculate joint hazard ratios (JHR) to assess the joint association of walkability and greenness on fracture.^29^ The CRI method has been widely used to evaluate the joint association of environmental exposures on health outcomes.^29,30,31^ We denoted the JHR according to the combination of the *p* environmental exposures as the CRI and defined it as:
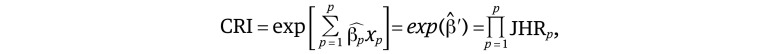
where β̂′ = (β̂_1_,…,β̂*_p_*) are the estimates of the log hazard ratio (HR) for the *p* exposures estimated in a Cox model consisting of all *p* exposures together. *x*′ = (*x*_1_,…,*x_p_*) are the levels at which each HR is evaluated, in our case an increase in IQR. *JHR_p_* = exp(β̂*_p_x_p_*) denotes the cumulative hazard ratio for the *p*^th^ exposure in a multi-exposure model. The 95% CI of CRI is defined by: 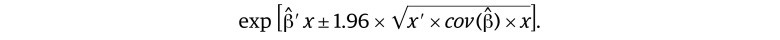
For estimating JHRs, we reversed the direction of the association of neighborhood walkability and residential greenness with fracture compared with the other analyses. Consequently, the JHR was defined as HR for an IQR decrease in walkability and greenness compared with no decrease in either of the 2 exposures.

To disentangle the potential effect modification of walkability upon the association of greenness and fracture, we also tested the interaction effect between walkability and greenness by a likelihood ratio test. We fitted a model involving a multiplicative interaction between 2-degree-of-freedom natural spline terms for walkability and NDVI (considering both as continuous variables). We plotted the regression surface using a contour plot to visualize the model according to the R visreg package.^32^ In addition, we also examined the association of greenness (considering it categorical variable) with fracture in different quarters of walkability areas.

Stratified analyses were performed by age at baseline (<65 or ≥65 years), sex (male or female), BMI (underweight [<18.5], normal weight [18.5 to <24.0], and overweight or obesity [≥24.0]; BMI is calculated as weight in kilograms divided by height in meters squared) and household income (<30 000 yuan per year or ≥30 000 yuan per year). We also built 2 models with or without an interaction term between the exposure and the modifier and used a likelihood ratio test to determine potential interactive effect.

We performed several sensitivity analyses to examine the robustness of our results. First, we repeated our main analysis with multiple imputation via chained equations to evaluate the outcomes of missing values of covariates. Second, we restricted our analyses to participants who did not relocate during the 10 years before baseline to account for exposure misclassification. Third, we altered the end point of follow-up to December 31, 2019, considering the potential influence of COVID-19 massive lockdowns. Fourthly, we extended the outcome to all types of fracture, including pathologic fracture, stress fracture, and injury fracture, to examine whether the associations were consistent in all types of fractures. Finally, we further excluded participants with a rheumatoid arthritis diagnosis at baseline, which may contribute to fracture.^33^ All analyses were performed in R version 4.1.1 (R Project for Statistical Computing) and a 2-tailed *P* value of less than .05 was defined as statistically significant. Data were analyzed in March 2023.

## Results

Overall, a total of 23 940 participants were included in the analysis. During 134 638 person-years of follow-up, a total of 3322 (13.9%) participants received a fracture diagnosis. Baseline characteristics are listed in Table 1. The mean (SD) age of the study participants was 63.4 (9.4) years, and 13 735 (57.4%) were female. Participants with fracture were more likely to be older, to be female, to have a lower BMI, and have a lower level of household income and education. Details of missing data for each variable are presented in eTable 2 in Supplement 1. Neighborhood walkability was moderately correlated with NDVI (Spearman ρ = −0.54; 95% CI, −0.53 to −0.55; *P* < .001).

**Table 1.** Baseline Characteristics of the Study Population

Characteristics	Patients, No (%)			*P* value^a^
	Fracture (n = 3322)	Nonfracture (n = 20 618)	Total (N = 23 940)	
Age at baseline, mean (SD), y	64.7 (9.0)	63.2 (9.5)	63.4 (9.4)	<.001
Sex				
Male	1118 (33.7)	9087 (44.1)	10 205 (42.6)	<.001
Female	2204 (66.3)	11 531 (55.9)	13 735 (57.4)	
Body mass index, mean (SD)^b^	20.4 (3.8)	20.9 (3.8)	20.8 (3.8)	<.001
Education				
Illiterate	1294 (39.0)	6801 (33.0)	8095 (33.8)	<.001
Primary or middle school	1955 (58.9)	13 057 (63.3)	15 012 (62.7)	
High school or above	73 (2.2)	760 (3.7)	833 (3.5)	
Occupation				
Industry or agriculture	1364 (41.1)	8805 (42.7)	10 169 (42.5)	.001
Enterprise or public institution	85 (2.6)	702 (3.4)	787 (3.3)	
Housework or retirement	1762 (53.0)	10 280 (49.9)	12 042 (50.3)	
Others	111 (3.3)	831 (4.0)	942 (3.9)	
Household income per year, CNY				
<10 000	226 (6.8)	1383 (6.7)	1609 (6.7)	<.001
10 000-29 999	1399 (42.1)	7539 (36.6)	8938 (37.3)	
30 000-49 999	970 (29.2)	5942 (28.8)	6912 (28.9)	
≥50 000	727 (21.9)	5754 (27.9)	6481 (27.1)	
History of osteoporosis				
Yes	1798 (54.1)	13 582 (65.9)	15 380 (64.2)	<.001
No	1524 (45.9)	7036 (34.1)	8560 (35.8)	
History of diabetes				
Yes	2683 (80.8)	17 306 (83.9)	19 989 (83.5)	<.001
No	639 (19.2)	3312 (16.1)	3951 (16.5)	
PM_2.5_, median (IQR)^c^	34.0 (31.4-37.5)	34.0 (31.4-37.5)	34.0 (31.4-37.5)	.78
Walkability, median (IQR)	11.7 (5.7-34.1)	11.6 (5.7-40.4)	11.7 (5.7-39.9)	.66
NDVI, median (IQR)^d^	0.4 (0.3-0.5)	0.4 (0.4-0.5)	0.4 (0.4-0.5)	.001

Abbreviations: CNY, Chinese yuan; NDVI, normalized difference vegetation index; PM_2.5_, particulate matter with an aerodynamic diameter ≤2.5 μm.

^a^ χ^2^ test was used for categorical variables and analysis of variance and Kruskal-Wallis test was used for continuous variables.

^b^ Body mass index is calculated as weight in kilograms divided by height in meters squared.

^c^ One-year average concentrations of air pollutants before the baseline.

^d^ NDVI was measured at a buffer of 1000 m.

**Figure 2.**
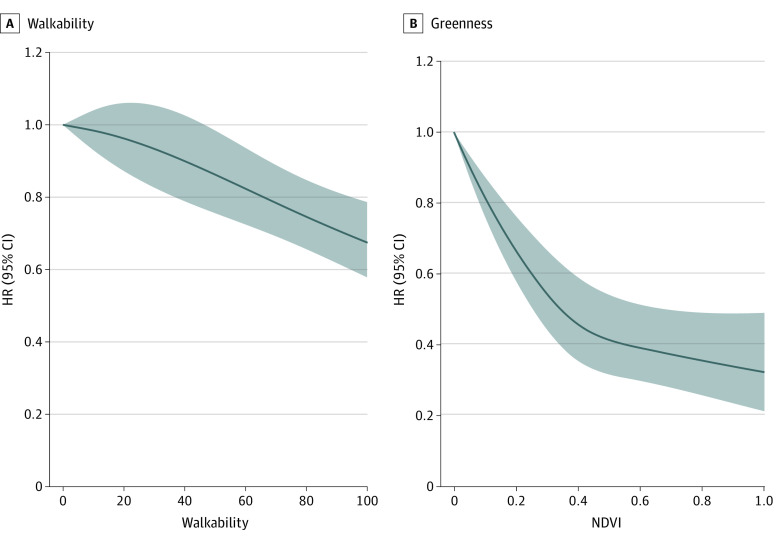
Multivariate-Adjusted Spline Curves for the Associations of Walkability and Greenness With Incident Fracture Adjusted for age (timescale), sex, household income, education, occupation, body mass index, history of osteoporosis, history of diabetes, and concentration of particulate matter with aerodynamic diameter ≤2.5 μm. Greenness was measured using a normalized difference vegetation index (NDVI) with a buffer of 1000 m. Solid line represents point estimate and shading represents 95% CI. HR indicates hazard ratio.

The associations of neighborhood walkability and residential greenness with fracture are summarized in Table 2. In the full adjusted model, every IQR increment in neighborhood walkability and residential greenness was associated with an HR of 0.88 (95% CI, 0.83-0.92) and 0.84 (95% CI, 0.80-0.89), respectively, for fracture. In model 1, higher greenness and walkability were both associated with lower risk of fracture. When further adjusted for sex, household income, education, occupation, BMI, history of osteoporosis, history of diabetes, and concentration of PM_2.5_, the associations remained stable with HRs of 0.96 (95% CI, 0.92-1.00) and 0.96 (95% CI, 0.92-0.99) for walkability and greenness per IQR increment, respectively. In the 2-exposure model, we found that the estimated associations for walkability and greenness with fracture were greater. Compared with individuals in the lowest Q of walkability, participants in the highest Q had a 23% (HR, 0.77; 95% CI, 0.69-0.86) reduced risk of fracture. Similarly, the HR of NDVI-1000 m changed from 0.96 (95% CI, 0.92-0.99) to 0.91 (95% CI, 0.86-0.95), per IQR increment. The exposure-response association for walkability and greenness from the RCS models are presented in Figure 2. Lower levels of walkability and greenness were associated with higher risk of incident fracture.

**Table 2.** Associations of Neighborhood Walkability and Residential Greenness With Risk of Fracture

Exposure	Cases/person-years	Adjusted HRs (95% CIs)		
		Model 1^a^	Model 2^b^	Model 3^c^
Walkability				
Q1 (0-5.75)	874/34 219	1 (Reference)	1 (Reference)	1 (Reference)
Q2 (5.76-11.68)	802/34 359	0.94 (0.85-1.03)	0.94 (0.85-1.04)	0.92 (0.83-1.01)
Q3 (11.69-39.93)	871/31 924	1.08 (0.99-1.19)	1.13 (1.03-1.25)	1.03 (0.93-1.15)
Q4 (39.94-100)	775/34 136	0.89 (0.81-0.99)	0.88 (0.79-0.97)	0.77 (0.69-0.86)
* P* for trend	NA	.24	.14	<.001
Per IQR increase	NA	0.96 (0.92-1.00)	0.96 (0.92-1.00)	0.95 (0.90-0.99)
Greenness				
Q1 (0.00-0.35)	961/33 751	1 (Reference)	1 (Reference)	1 (Reference)
Q2 (0.36-0.40)	809/34 290	0.86 (0.78-0.94)	0.83 (0.75-0.91)	0.82 (0.75-0.91)
Q3 (0.41-0.48)	696/32 553	0.80 (0.72-0.88)	0.78 (0.70-0.86)	0.74 (0.66-0.82)
Q4 (0.49-0.79)	856/34 044	0.90 (0.82-0.99)	0.83 (0.74-0.92)	0.74 (0.66-0.84)
* P* for trend	NA	.010	<.001	<.001
Per IQR increase	NA	0.96 (0.92-0.99)	0.96 (0.92-0.99)	0.91 (0.86-0.95)

Abbreviations: HR, hazard ratio; NDVI, normalized difference vegetation index; PM_2.5_, particulate matter with aerodynamic diameter ≤2.5 μm; Q, quartile.

^a^ Model 1 was adjusted for age (timescale).

^b^ Model 2 was further adjusted for sex, household income, education, occupation, body mass index, history of osteoporosis, history of diabetes, and concentration of PM_2.5_.

^c^ Model 3 was further adjusted for NDVI or walkability.

As for CRI analyses (eFigure 1 in Supplement 1), we found that the association estimate for joint exposure of decreased walkability and decreased NDVI (JHR, 1.35; 95% CI, 1.24-1.48) was greater than the HR for single exposure. We observed that with the increase of greenness, the risk of incident fracture was greater in neighborhoods with lower walkability (eFigure 2 in Supplement 1). In the highest Q (Q4) of walkability, the HR for the highest Q (Q4) of greenness was 0.62 (95% CI, 0.46-0.82) as compared with the lowest Q (Q1) of greenness (eFigure 3 in Supplement 1). However, the interaction term between walkability and greenness (HR, 0.95; 95% CI, 0.89-1.01; *P* for interaction = .11) was not statistically significant.

**Figure 3.**
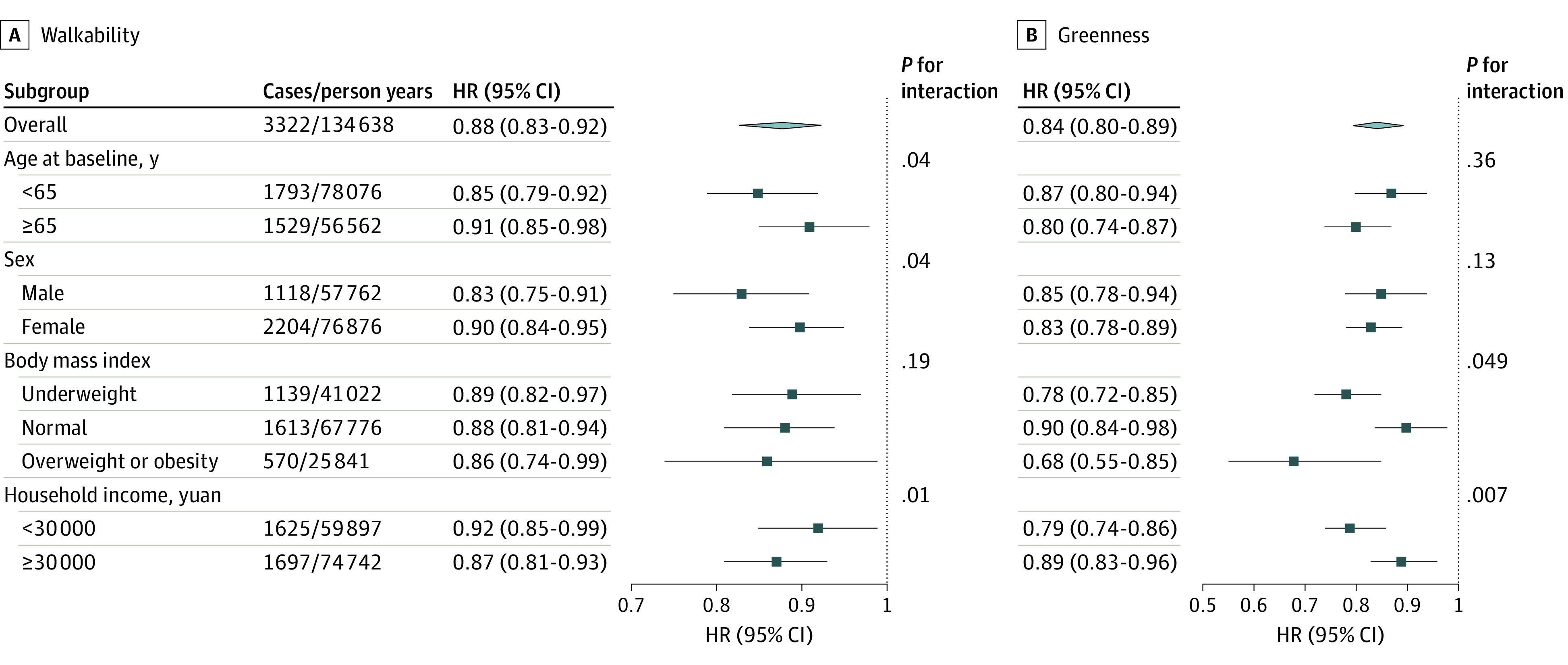
Stratified Analysis for the Associations of Walkability and Greenness With Incident Fracture Adjusted for age (timescale), sex, household income, education, occupation, body mass index, history of osteoporosis, history of diabetes, and concentration of particulate matter with aerodynamic diameter of ≤2.5 μm or smaller. Greenness was measured using a normalized difference vegetation index with a buffer of 1000 m. Results were presented as hazard ratio (HRs) with corresponding 95% CIs for per IQR increase in walkability or greenness.

Results of stratified analyses are shown in Figure 3. Greater associations of walkability and fracture were observed among younger participants, men, or participants with higher levels of income, whereas greater associations were observed for greenness among participants with low levels of income or higher BMI.

Sensitivity analyses by performing multiple chained imputation for missing covariates, excluding participants who had moved during the 10 years before baseline or altering the end point of follow-up to December 31, 2019, did not materially alter our results (eTable 3, eTable 4, and eTable 5 in Supplement 1). When defining our outcome as all types of fracture or excluding participants with a rheumatoid arthritis diagnosis, the results remained consistent with the main analysis (eTable 6 and eTable 7 in Supplement 1).

## Discussion

Long-term exposure to higher levels of walkability and greenness were both associated with decreased risk of incident fracture. Moreover, we observed that the benefits of greenness on fracture increased as the level of walkability increased.

To the best of our knowledge, this is the first attempt to investigate the associations of exposure to neighborhood walkability with incident fracture in a population-based prospective cohort study. Previous evidence on the association between greenness and bone health was very scarce. Only 2 relevant studies were identified, but their findings were contradictory. In line with our findings, a cross-sectional study in China suggested higher residential greenness was significantly associated with an increase in calcaneus quantitative ultrasound index (an indicator of bone strength) with a change of 3.57 (95% CI, 3.34-3.80) for every IQR increase in NDVI-1000 m.^23^ However, a prospective study in Hong Kong, China revealed that participants living near higher green spaces had higher risk of incident fracture, which was inconsistent with the proposed hypothesis that green space may contribute to bone health, thereby reducing the risk of fracture.^24^

Although the biological pathways have not been yet fully clarified, the observed associations may be explained by physical activity-based mechanisms.^17,18,34^ Prior studies have demonstrated that walkable attributes of built environment could predict active leisure activities and walking, even when adjusted for subjective preferences.^35,36^ Green spaces could provide opportunities for routine and recreational physical activity, promote physical activity and, in turn, improve bone strength, reduce fall risk, and potentially prevent the occurrence of osteoporosis.^37,38^ In addition, accumulating epidemiological evidence suggested that physical activity was positively associated with increased bone mineral density, retarded bone loss, and decreased risk of fracture.^39,40^ Association of physical activity and fracture may be elucidated by the following biological pathways. Physical activity acts as mechanical stimuli that stimulates bone growth and remodeling through the osteocyte response to biomechanical load.^41^ Mechanical forces, which were exerted on the bones during physical activity, induce the maintenance or gain of bone mass and drive adaptation of bone structure.^42,43^ Physical activity also affects lipid metabolism and body composition and stimulates endocrine glands to regulate the concentration and absorption of vitamin D and serum calcium, thereby promoting bone mineral density.^44,45,46^ Furthermore, regular physical activity could promote increased exposure to outdoor sunshine and promote calcium absorption. Studies also indicated that decreased concentration of air pollution in greener areas was associated with healthier bones.^20,21,47^ Consistent with our study, emerging evidence suggested inverse correlation between walkability and greenness.^48,49^ The level of physical activity promoted by high greenness may be partly enhanced by high walkability. Moreover, an elevated level of walkability in residential areas may heighten the likelihood of air pollution exposure, but this risk could be potentially mitigated by the presence of greenness.^50^ In addition, our findings may be explained by the stress-reducing properties of residential areas with more greenery and walkability.

### Strengths and Limitations

Our study has several strengths. First, as far as we know, this is the first investigation of the independent and joint association of both neighborhood walkability and residential greenness with risk of fracture. In addition, our study benefited from a longitudinal design, large sample size, and abundant information on covariates. Second, linkage to YHIS allowed us to follow up efficiently and obtain medical records (including date of incident fracture and history of disease) accurately, which could avoid recall bias. Third, we controlled for potential confounding bias of air pollution. Previous studies^21,51^ have suggested the correlations between air pollution, neighborhood walkability, and residential greenness. Fourthly, we adopted objective measures to assess exposure variables, thus avoiding potential bias caused by subjective feelings of participants. The developed Walk Score tool could provide a standardized measurement of walkability for land use, reducing subjectivity and uncertainty.^52^

Some limitations also exist that need further elaboration. First, we assessed walkability and greenness only according to residential address and did not consider the exposure levels of other locations (eg, workplace) or duration spent at different locations. Previous studies^46,53^ have shown that the assessment of walkability and greenness only according to residential address could result in weaker correlation with physical activity, thereby underestimating the associations of walkability and greenness with fracture. When interpreting our findings, a lack of temporal change of walk score must also be considered. Second, relocating after baseline may introduce misclassification bias by altering the exposure levels. Although we lack data on relocating after baseline, we repeated our main analysis among participants who did not relocate during the 10 years before baseline and the results of the sensitivity analysis did not change substantially. Third, due to lack of data on physical activity, we could not explore the mediation effect of physical activity. Additionally, participants recruited in our study lived in the Yinzhou district, Ningbo, Zhejiang Province, which may limit the generalizability of our findings. Further studies in other populations are needed to confirm our findings.

## Conclusions

In this cohort study of more than 20 000 participants, we found that exposure to higher levels of neighborhood walkability and residential greenness were both associated with lower risk of incident fracture. Furthermore, we observed a greater association of residential greenness and fracture in more walkable areas. Our findings are of great value for urban planners in designing neighborhoods with adequate greenness and walking-friendly amenities to encourage physical activity and ultimately decrease risk of fracture.

## Notes

Supplement 1.
eMethods.
**eFigure 1. **Joint Hazard Ratios for Associations of Decreased Neighborhood Walkability and Decreased Residential Greenness With Incident of Fracture **eFigure 2. **Contour Plot for the Interaction Effect of Walkability and Greenness on Incident Fracture **eFigure 3. **Associations Between Quartiles of Greenness and Incident Fracture by Walkability Quartiles **eTable 1. **Number of Fractures by Different Sites and ICD-10 Codes **eTable 2. **Number of Participants With Missing Data for Each Variable of Interest **eTable 3. **Sensitivity Analyses by Using Multiple Imputation Chained Equations to Impute Missing Values of Covariates **eTable 4. **Sensitivity Analyses by Excluding Participants Who had Relocated Within 10 Years Before Baseline **eTable 5. **Sensitivity Analyses by Altering the End Point of Follow-Up to December 31, 2019 **eTable 6. **Sensitivity Analyses by Including Pathologic Fractures, Stress Fractures and Injury Fractures **eTable 7. **Sensitivity Analyses by Excluding Participants Who Received a Rheumatoid Arthritis Diagnosis at Baseline
eReferences

Supplement 2.
Data Sharing Statement
