# Supplementary material for: Exposure to Neighborhood Walkability and Residential Greenness and Incident Fracture
Source: JAMA Netw Open. 2023 Sep 28;6(9):e2335154. doi: 10.1001/jamanetworkopen.2023.35154 (PMC10539990; doi:10.1001/jamanetworkopen.2023.35154)
Supplement: Supplement 1. — eMethods. eFigure 1. Joint Hazard Ratios for Associations of Decreased Neighborhood Walkability and Decreased Residential Greenness With Incident of Fracture eFigure 2. Contour Plot for the Interaction Effect of Walkability and Greenness on Incident Fracture eFigure 3. Associations Between Quartiles of Greenness and Incident Fracture by Walkability Quartiles eTable 1. Number of Fractures by Different Sites and ICD-10 Codes eTable 2. Number of Participants With Missing Data for Each Variable of Interest eTable 3. Sensitivity Analyses by Using Multiple Imputation Chained Equations to Impute Missing Values of Covariates eTable 4. Sensitivity Analyses by Excluding Participants Who had Relocated Within 10 Years Before Baseline eTable 5. Sensitivity Analyses by Altering the End Point of Follow-Up to December 31, 2019 eTable 6. Sensitivity Analyses by Including Pathologic Fractures, Stress Fractures and Injury Fractures eTable 7. Sensitivity Analyses by Excluding Participants Who Received a Rheumatoid Arthritis Diagnosis at Baseline eReferences [file jamanetwopen-e2335154-s001.pdf]

Supplemental Online Content

Zhu Z, Yang Z, Xu L, et al. Exposure to neighborhood walkability and residential greenness with incident fracture. *JAMA Netw Open*. 2023;6(9):e2334266. doi:10.1001/jamanetworkopen.2023.34266

eMethods.
eFigure 1. Joint Hazard Ratios for Associations of Decreased Neighborhood Walkability and Decreased Residential Greenness With Incident of Fracture
eFigure 2. Contour Plot for the Interaction Effect of Walkability and Greenness on Incident Fracture
eFigure 3. Associations Between Quartiles of Greenness and Incident Fracture by Walkability Quartiles
eTable 1. Number of Fractures by Different Sites and ICD-10 Codes
eTable 2. Number of Participants With Missing Data for Each Variable of Interest
eTable 3. Sensitivity Analyses by Using Multiple Imputation Chained Equations to Impute Missing Values of Covariates
eTable 4. Sensitivity Analyses by Excluding Participants Who had Relocated Within 10 Years Before Baseline
eTable 5. Sensitivity Analyses by Altering the End Point of Follow-Up to December 31, 2019
eTable 6. Sensitivity Analyses by Including Pathologic Fractures, Stress Fractures and Injury Fractures
eTable 7. Sensitivity Analyses by Excluding Participants Who Received a Rheumatoid Arthritis Diagnosis at Baseline
eReferences

This supplemental material has been provided by the authors to give readers additional information about their work.

© 2023 Zhu Z et al. *JAMA Network Open.*

eMethods.

Exposure: Neighborhood Walkability

Walk Score (https://www.walkscore.com/), measured by geographic information systems (GIS), was used to quantify neighborhood walkability of each participant. Several studies have demonstrated the validation of Walk Score for estimating neighborhood walkability[1,2]. Based on the Walk Score framework, Su and colleagues proposed a modified walkability calculation method suitable for neighborhoods in China[3,4]. Details of the methodology have been elaborated in previous studies[3,4]. In brief, the assessment process was mainly carried out in four steps:

1. Point of interest (POI) selection and weights determination. We selected 6 principle amenities (19 items) and their weight from Su's study[3]. Then, we identified amenities in July 2015 around each participant's neighborhood via road network files through Gaode Map (https://lbs.amap.com/api/, accessed August 26, 2021).
2. Travel time calculation and decay function determination. Considering the actual road network and traffic condition, we used Gaode Map to calculate the travel time from residence address to each POI. We adopted decay function to reflect that residents' interest in POI would gradually decay with the increase of travel time. We utilized the tolerance time approach, as demonstrated in Walk Score (https://www.walkscore.com/), to estimate the decay function. Combining the decay function and the travel time, we obtained the adjusted weight of POI. The decay function was demonstrated as follows:

© 2023 Zhu Z et al. *JAMA Network Open.*

$$f(t) = \begin{cases} 1, t \le 5\ \text{min} \\ -0.0007867177 \times t^3 + 0.0268454656 \times t^2 - 0.31677648 \times t + 2.011086, 5 < t \le 20\ \text{min} \\ -0.00047514 \times t^3 + 0.0362624 \times t^2 - 0.92248 \times t + 7.8671, 20 < t \le 30\ \text{min} \\ 0, t > 30\ \text{min} \end{cases}$$

3. Original walk score calculation and adjustment. We summed the weights of each POI to obtain the original walk score. We further adjusted for pedestrian characteristics, which were identified by ArcGIS software (V 10.6) to obtain adjusted walk score. The decay rate for the pedestrian characteristic factors was presented in Su's study[3]. The calculation of adjusted walk score was presented as follows:

$$\text{Adjusted walk score} = \text{Original walk score} \times (1 - \text{decay rate of intersection density}) \times (1 - \text{decay rate of block length})$$

4. Standardization of walk score. Walk score was standardized into the interval between 0 and 100, which was assigned to each participant according to the address. The standardization formula was presented as follows:

$$\text{Standardized walk score} = \frac{\text{Adjusted walk score} - \text{min (adjusted walks core)}}{\text{max(adjusted walk score)} - \text{min (adjusted walk score)}}$$

Exposure: Residential Greenness

Exposure to residential greenness was assessed by satellite-derived Normalized Difference Vegetation Index (NDVI). NDVI, an indicator of greenness quality and intensity, was measured based on differential spectral signatures of chlorophyll in the visible (red band) and near infrared regions[5]. NDVI was calculated as the ratio of the

© 2023 Zhu Z et al. *JAMA Network Open.*

difference of near-infrared region and red reflectance to the sum of these two measures[6]. The NDVI values ranged from -1 to 1, with higher values indicating a higher level of greenness. Data on greenness were obtained from the moderate resolution imaging spectroradiometer (MODIS) (https://modis.gsfc.nasa.gov/data/dataprod/mod13.php) carried in Terra and Aqua satellites launched by the National Aeronautics and Space Administration (NASA)[7,8]. Imageries on greenness with a spatial resolution were provided by MODIS for every 16 days. We calculated NDVI based on the same days of the year (days 001, 017, 097, 113, 193, 209, 257, 289, and 305) to represent the four seasons[9]. We linked the imageries to the residential address of each participant and NDVI was calculated in radii of 1000m around residential address based on ArcGIS. In this study, one-year average NDVI before the baseline were calculated as an indicator of residential greenness.

Covariates

All covariates were measured at baseline, including: age at baseline (in years), gender (males or females), body mass index (BMI), education (illiterate, primary or middle school, and high school or above), occupation (industry or agriculture, enterprise or public institution, housework or retirement, and others), household income (<10,000, 10,000–29,999, 30,000–49,999, and ≥50,000 Chinese Yuan (CNY) per year), history of osteoporosis (yes or no), history of diabetes (yes or no) and concentration of $PM_{2.5}$. Demographic information and lifestyle factors were obtained using standardized questionnaires by trained healthcare staff at baseline. Age at baseline was calculated as the time interval between date of birthday and baseline. We categorized participants

© 2023 Zhu Z et al. *JAMA Network Open.*

into three 3 groups: underweight (BMI $<$18.5 kg/m2), normal weight (18.5 ≤ BMI $<$ 24.0 kg/m^2) and overweight or obesity (24.0 kg/m^2 ≤ BMI) according to the guidelines for prevention and control of overweight and obesity in Chinese adults[10]. History of osteoporosis or diabetes was defined using ICD-10 codes (E10-E14; M80-M82) based on medical records through the YHIS at baseline. We calculated 1-year average concentrations of $PM_{2.5}$ by land-use regression model before the baseline for each participant[11].

© 2023 Zhu Z et al. *JAMA Network Open.*

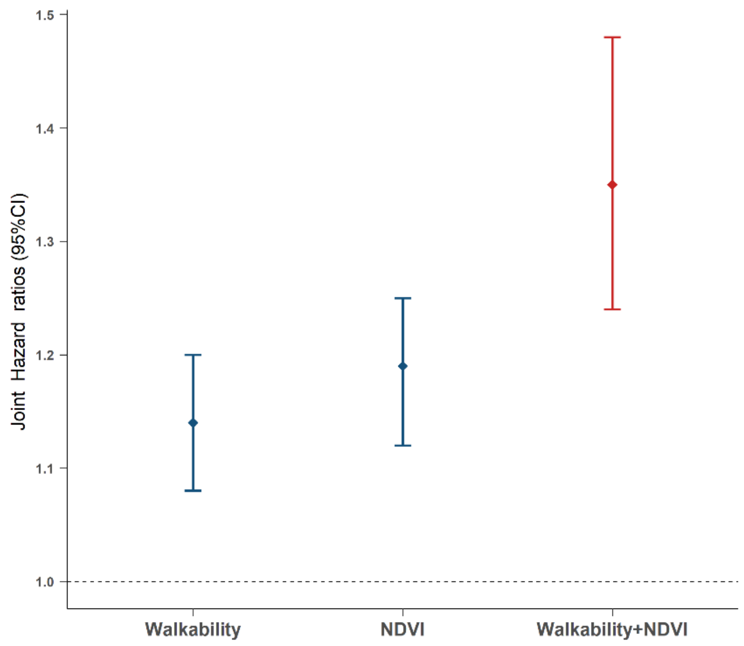

eFigure 1. Joint Hazard Ratios for Associations of Decreased Neighborhood Walkability and Decreased Residential Greenness With Incident of Fracture.

Abbreviation: NDVI, normalized difference vegetation index; BMI, body mass index; $PM_{2.5}$, particulate matter with aerodynamic diameter ≤ 2.5 μm. The joint hazard ratios (JHR) were represented per IQR decrease in walkability and IQR decrease in NDVI relative to no decrease in either of two exposures.

IQR for walkability: 34.18 , NDVI: 0.13.

Adjusted for age (timescale), gender, household income, education, occupation, BMI, history of osteoporosis, history of diabetes and concentration of $PM_{2.5}$.

NDVI was measured at a buffer of 1000 m.

© 2023 Zhu Z et al. *JAMA Network Open.*

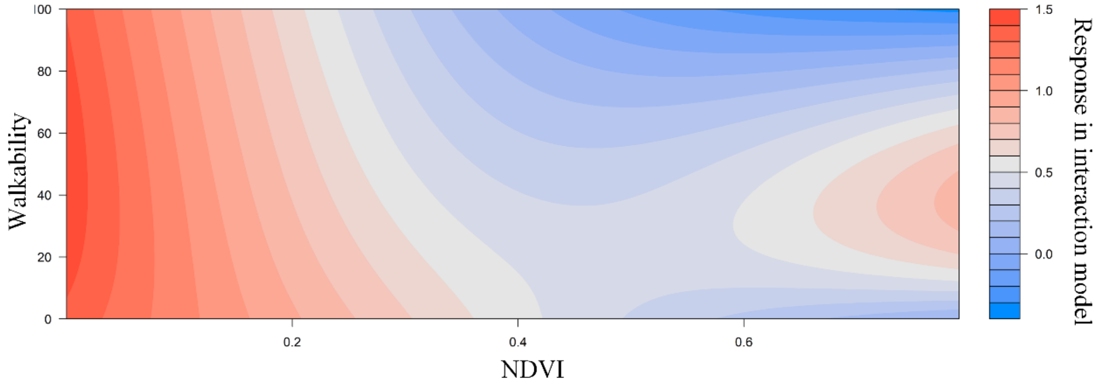

eFigure 2. Contour Plot for the Interaction Effect of Walkability and Greenness on Incident Fracture.
Abbreviation: NDVI, normalized difference vegetation index; BMI, body mass index; $PM_{2.5}$, particulate matter with aerodynamic diameter $\leq$ 2.5 μm.
Adjusted for age (timescale), gender, household income, education, occupation, BMI, history of osteoporosis, history of diabetes and concentration of $PM_{2.5}$.
NDVI was measured in buffer of 1000 m.

© 2023 Zhu Z et al. *JAMA Network Open.*

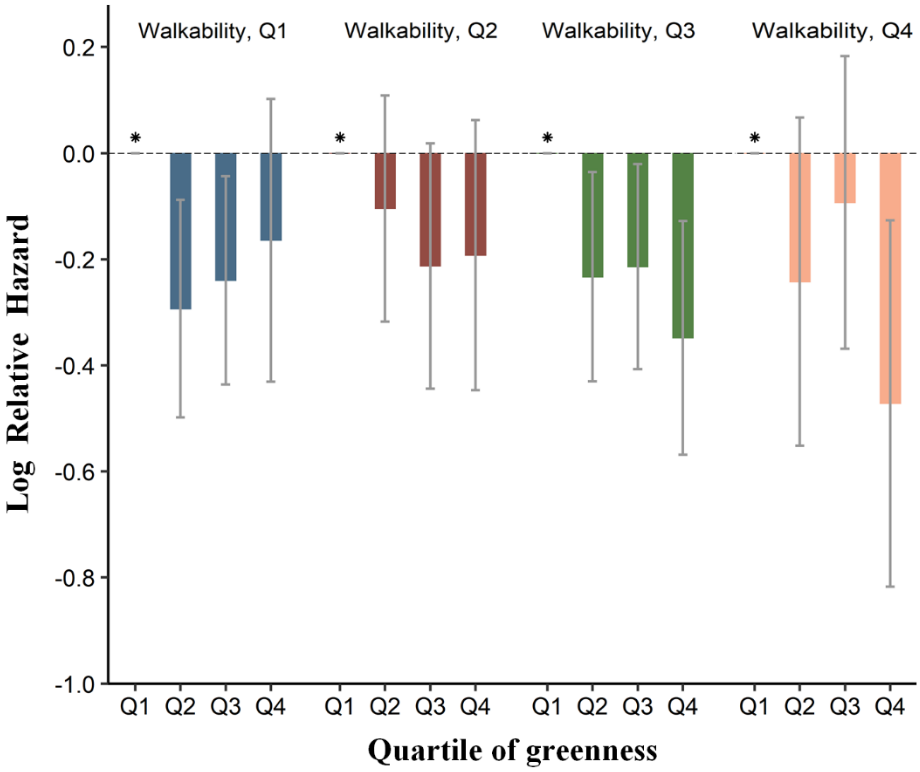

eFigure 3. Associations Between Quartiles of Greenness and Incident Fracture by Walkability Quartiles.

Abbreviation: HR, hazard ratio; CI, confidence interval; Q, quartile; $PM_{2.5}$, particulate matter with aerodynamic diameter ≤ 2.5 μm; BMI, body mass index; NDVI, normalized difference vegetation index.

Adjusted for age (timescale), sex, household income, education, occupation, BMI, history of osteoporosis, history of diabetes, concentration of $PM_{2.5}$.

Greenness was measured using NDVI in buffer of 1000m.

© 2023 Zhu Z et al. *JAMA Network Open.*

eTable 1. Number of Fractures by Different Sites and *ICD-10* Codes

Site	ICD-10 code	n (% of fractures)
Skull and face	S02	114(3.4)
Neck	S12	10(0.3)
Ribs, sternum and thoracic vertebra	S22	973(29.3)
Lumbar vertebra and pelvis	S32	485(14.6)
Shoulder and upper arm	S42	216(6.5)
Forearm	S52	417(12.6)
Wrist and hand	S62	229(6.9)
Femur	S72	176(5.3)
Knee, lower leg and ankle	S82	377(11.4)
Feet	S92	325(9.8)

© 2023 Zhu Z et al. *JAMA Network Open.*

eTable 2. Number of Participants With Missing Data for Each Variable of Interest [a]

Variables with missing data	n (%) [b]
**Total at baseline**	32097
**Residential greenness**	
No missing	32035(99.81)
Missing	62(0.19)
**BMI, n(%)**	
No missing	32033(99.80)
Missing	64(0.20)
**Occupation, n(%)**	
Industry or agriculture	13079(40.80)
Enterprise or public institution	1288(4.01)
Housework or retirement	16046(49.99)
Others	1359(4.23)
Missing	325(1.01)
**Household income per year, n (%)**	
<10,000	2080(6.48)
10,000-29,999	11356(35.38)
30,000-49,999	9132(28.45)
≥50,000	9296(28.96)
Missing	233(0.73)
**Education, n(%)**	
Illiterate	10413(32.44)
Primary or middle school	19692(61.35)
High school or above	1852(5.77)
Missing	140(0.44)

Abbreviations: BMI, body mass index.

[a] Participants who were lost to follow-up and those with unidentified addresses or ID or duplicated records were excluded.

[b] Values were presented as number (percentages).

© 2023 Zhu Z et al. *JAMA Network Open.*

eTable 3. Sensitivity Analyses by Using Multiple Imputation Chained Equations to Impute Missing Values of Covariates

Exposure	Cases/Person-years	Adjusted HRs(95%CIs)
Walkability		
Q1	901/35404	1.00 (Ref)
Q2	814/34904	0.90(0.82, 1.02)
Q3	896/32805	1.04(0.94, 1.14)
Q4	793/35014	0.77(0.66, 0.88)
P for trend		<0.001
Per IQR increment		0.88(0.83, 0.93)
Greenness		
Q1	982/34627	1.00 (Ref)
Q2	829/35101	0.83(0.73, 0.93)
Q3	712/33263	0.74(0.64, 0.84)
Q4	881/35136	0.74(0.62, 0.86)
P for trend		<0.001
Per IQR increment		0.85(0.79, 0.90)

Abbreviations: HR, hazard ratio; CI, confidence interval; Q, quartile; Ref, reference; IQR, interquartile range; NDVI, normalized difference vegetation index; BMI, body mass index; $PM_{2.5}$, particulate matter with aerodynamic diameter ≤ 2.5 µm.

[a] Adjusted for age (timescale), gender, household income, education, occupation, BMI, history of osteoporosis, history of diabetes and concentration of $PM_{2.5}$.

© 2023 Zhu Z et al. *JAMA Network Open.*

eTable 4. Sensitivity Analyses by Excluding Participants Who had Relocated Within 10 Years Before Baseline

Exposure	Cases/Person-years	Adjusted HRs(95%CIs)
Walkability		
Q1	859/33509	1.00 (Ref)
Q2	800/34150	0.91 (0.83, 1.01)
Q3	834/30687	1.02 (0.92, 1.14)
Q4	755/33195	0.76 (0.68, 0.85)
P for trend		<0.001
Per IQR increment		0.87 (0.83, 0.92)
Greenness (NDVI)		
Q1	898/31446	1.00 (Ref)
Q2	803/33940	0.82 (0.74, 0.90)
Q3	695/32306	0.73 (0.66, 0.81)
Q4	852/33849	0.74 (0.65, 0.83)
P for trend		<0.001
Per IQR increment		0.84 (0.79, 0.89)

Abbreviations: HR, hazard ratio; CI, confidence interval; Q, quartile; Ref, reference; IQR, interquartile range; NDVI, normalized difference vegetation index; BMI, body mass index; $PM_{2.5}$, particulate matter with aerodynamic diameter ≤ 2.5 μm.

[a] Adjusted for age (timescale), gender, household income, education, occupation, BMI, history of osteoporosis, history of diabetes and concentration of $PM_{2.5}$.

© 2023 Zhu Z et al. *JAMA Network Open.*

eTable 5. Sensitivity Analyses by Altering the Endpoint of Follow-Up to December 31, 2019

Exposure	Cases/Person-years	Adjusted HRs(95%CIs)
Walkability		
Q1	360/17593	1.00 (Ref)
Q2	332/17475	0.96 (0.82, 1.11)
Q3	349/16167	1.08 (0.91, 1.27)
Q4	334/17728	0.82 (0.69, 0.97)
P for trend		0.058
Per IQR increment		0.88 (0.81, 0.95)
Greenness (NDVI)		
Q1	397/17672	1.00 (Ref)
Q2	337/17262	0.85 (0.73, 0.99)
Q3	259/16008	0.71 (0.60, 0.85)
Q4	382/18019	0.76 (0.63, 0.92)
P for trend		<0.001
Per IQR increment		0.84 (0.77, 0.91)

Abbreviations: HR, hazard ratio; CI, confidence interval; Q, quartile; Ref, reference; IQR, interquartile range; NDVI, normalized difference vegetation index; BMI, body mass index; $PM_{2.5}$, particulate matter with aerodynamic diameter ≤ 2.5 μm.

[a] Adjusted for age (timescale), gender, household income, education, occupation, BMI, history of osteoporosis, history of diabetes and concentration of $PM_{2.5}$.

© 2023 Zhu Z et al. *JAMA Network Open.*

eTable 6. Sensitivity Analyses by Including Pathologic Fractures, Stress Fractures and Injury Fractures

Exposure	Cases/Person-years	Adjusted HRs(95%CIs)
Walkability		
Q1	936/30306	1.00 (Ref)
Q2	908/30460	0.98 (0.90, 1.08)
Q3	909/28881	1.05 (0.95, 1.17)
Q4	907/30560	0.87 (0.78, 0.97)
P for trend		0.636
Per IQR increment		0.93 (0.89, 0.98)
Greenness (NDVI)		
Q1	999/30106	1.00 (Ref)
Q2	897/30903	0.85 (0.78, 0.94)
Q3	780/28865	0.81 (0.73, 0.89)
Q4	984/30334	0.82 (0.73, 0.92)
P for trend		<0.001
Per IQR increment		0.89 (0.85, 0.94)

Abbreviations: HR, hazard ratio; CI, confidence interval; Q, quartile; Ref, reference; IQR, interquartile range; NDVI, normalized difference vegetation index; BMI, body mass index; $PM_{2.5}$, particulate matter with aerodynamic diameter ≤ 2.5 μm.

[a] Adjusted for age (timescale), gender, household income, education, occupation, BMI, history of osteoporosis, history of diabetes and concentration of $PM_{2.5}$.

© 2023 Zhu Z et al. *JAMA Network Open.*

eTable 7. Sensitivity Analyses by Excluding Participants Who Received a Rheumatoid Arthritis Diagnosis at Baseline

Exposure	Cases/Person-years	Adjusted HRs(95%CIs)
Walkability		
Q1	850/33500	1.00 (Ref)
Q2	790/33733	0.93 (0.84, 1.02)
Q3	852/31201	1.04 (0.93, 1.15)
Q4	752/33452	0.77 (0.69, 0.87)
P for trend		<0.001
Per IQR increment		0.88 (0.83, 0.92)
Greenness		
Q1	937/33148	1.00 (Ref)
Q2	790/33467	0.83 (0.75, 0.92)
Q3	681/31404	0.75 (0.67, 0.83)
Q4	836/33867	0.74 (0.66, 0.84)
P for trend		<0.001
Per IQR increment		0.84 (0.80, 0.89)

Abbreviations: HR, hazard ratio; CI, confidence interval; Q, quartile; Ref, reference; IQR, interquartile range; NDVI, normalized difference vegetation index; BMI, body mass index; $PM_{2.5}$, particulate matter with aerodynamic diameter $\leq$ 2.5m.

[a] Adjusted for age (timescale), gender, household income, education, occupation, BMI, history of osteoporosis, history of diabetes and concentration of $PM_{2.5}$.

© 2023 Zhu Z et al. *JAMA Network Open.*

eReferences

1. Duncan DT, Aldstadt J, Whalen J, Melly SJ, Gortmaker SL. Validation of Walk Score® for Estimating Neighborhood Walkability: An Analysis of Four US Metropolitan Areas. *International Journal of Environmental Research and Public Health*. 2011;8(11):4160-4179. doi:10.3390/ijerph8114160

2. Carr LJ, Dunsiger SI, Marcus BH. Validation of Walk Score for estimating access to walkable amenities. *Brit J Sport Med*. 2011;45(14):1144-1148. doi:10.1136/bjsm.2009.069609

3. Su S, Pi J, Xie H, Cai Z, Weng M. Community deprivation, walkability, and public health: Highlighting the social inequalities in land use planning for health promotion. *Land Use Policy*. 2017;67:315-326. doi:10.1016/j.landusepol.2017.06.005

4. Weng M, Ding N, Li J, et al. The 15-minute walkable neighborhoods: Measurement, social inequalities and implications for building healthy communities in urban China. *J Transp Health*. 2019;13:259-273. doi:10.1016/j.jth.2019.05.005

5. Crouse DL, Pinault L, Balram A, et al. Urban greenness and mortality in Canada's largest cities: a national cohort study. *The Lancet. Planetary Health*. 2017;1(7):e289-e297. doi:10.1016/S2542-5196(17)30118-3

6. Rhew IC, Vander Stoep A, Kearney A, Smith NL, Dunbar MD. Validation of the Normalized Difference Vegetation Index as a Measure of Neighborhood Greenness. *Ann Epidemiol*. 2011;21(12):946-952. doi:10.1016/j.annepidem.2011.09.001

7. Yu L, Li T, Yang Z, et al. Long-term exposure to residential surrounding greenness and incidence of diabetes: A prospective cohort study. *Environ Pollut*. 2022;310:119821. doi:10.1016/j.envpol.2022.119821

8. Yu Z, Wei F, Zhang X, et al. Air pollution, surrounding green, road proximity and Parkinson's disease: A prospective cohort study. *Environ Res*. 2021;197:111170. doi:10.1016/j.envres.2021.111170

9. Ji JS, Zhu A, Bai C, et al. Residential greenness and mortality in oldest-old women and men in China: a longitudinal cohort study. *The Lancet Planetary Health*. 2019;3(1):e17-e25. doi:https://doi.org/10.1016/S2542-5196(18)30264-X

10. Yang Z, Shen P, Qu Y, et al. Baseline and longitudinal trajectories of body-mass index and all-cause mortality among patients with type 2 diabetes. *Diabetes Metab*. 2023;49(3):101426. doi:10.1016/j.diabet.2023.101426

11. Zhang Z, Wang J, Hart JE, et al. National scale spatiotemporal land-use regression model for PM2.5, PM10 and NO2 concentration in China. *Atmos Environ*. 2018;192:48-54. doi:10.1016/j.atmosenv.2018.08.046

© 2023 Zhu Z et al. *JAMA Network Open.*
