# Supplementary material for: Exposure to Neighborhood Walkability and Residential Greenness and Incident Fracture
Source: JAMA Netw Open. 2023 Sep 28;6(9):e2335154. doi: 10.1001/jamanetworkopen.2023.35154 (PMC10539990; doi:10.1001/jamanetworkopen.2023.35154)
Supplement: Supplement 2. — Data Sharing Statement [file jamanetwopen-e2335154-s002.pdf]

Data Sharing Statement

Zhu. Exposure to Neighborhood Walkability and Residential Greenness With Incident Fracture. *JAMA Netw Open*. Published September 28, 2023. doi:10.1001/jamanetworkopen.2023.35154

Data
Data available: No
